# 

**Published:** 1900-07-28

**Authors:** 


### NOTICE TO CORRESPONDENTS.

All MS., Letters, Books for Review, and other matters intended for the Editor should be addressed The Editor, "The Hospital," The Hospital Building, 28 & 29, Southampton Street, Strand, W.C.

The Editor cannot undertake to return rejected MS., even when accompanied by stamped directed envelope.

**Notice to Advertisers and Subscribers.**

All Advertisements, Orders for Copies of The Hospital, and Business Communications should be addressed to **THE MANAGER (Not to the Editor)**, The Hospital, The Hospital Building, 28 & 29, Southampton Street, Strand, London, W.C.

The Cost of Subscription, post free, is as follows:—Per week, 2½d.; per quarter, 2s. 8d.; per half-year, 5s. 3d.; per annum, 10s. 6d. Foreign:—Per annum, 15s. 2d., payable, in all cases, in advance.

**NOTICE.—Vol. XXVII. of "The Hospital" (October, 1899, to April, 1900), in handsome cloth binding, is now on sale at the Office of this Journal, price 7s. 6d. Cases for binding the Numbers contained in this Volume 1s. 6d. Index to Vol. XXVII., 2½d., post free. The Monthly Part for August will be ready on August 1st. Price 6d., by post 8d.**

### The Student of Medicine.

### APPOINTMENTS.

W. Astin, M.B., C.M.Aberd., appointed Medical Officer of Health for the Loughton Urban District. J. H. Bellamy, M.R.C.S., L.R.C.P., appointed Senior Resident Medical Officer to the Sheffield Union Infirmary. G. Burgess, M.B., C.M.Aberd., appointed Medical Officer of Health for the Great Driffield Urban District. F. P. S. Creswell, B.Sc., B.S.Lond., F.R.C.S.Eng., appointed Assistant Ophthalmic Surgeon to the Cardiff Infirmary. R. B. Ferguson, M.D., appointed Certifying Factory Surgeon for the New Southgate District. G. H. Goldsmith, M.D.Cantab., appointed Medical Officer to the Bedford Union Workhouse. W. G. Gray, L.S.A., appointed Medical Officer of Health for the Holsworthy Urban District. W. Howells, M.D., C.M.Glasg., appointed Medical Officer to the Brecon Union Workhouse. H. J. Hutchens, M.R.C.S.,L.R.C.P., appointed Medical Superintendent of the Plague Hospital, Brisbane, Queensland. Eva McCall, M.B., Ch.B.Glasg., appointed Assistant Resident Medical Officer to the Infirmary of Salford Union. J. Mackeith, M.B., C.M., appointed Surgeon to the Exeter Dispensary. R. Pickard, M.S., M.D.Lond., appointed Surgeon to the Exeter Dispensary. J. E. Platt, M.S., M.D.Lond., F.R.C.S.Eng., appointed Honorary Assistant Surgeon to the Manchester Royal Infirmary. T. J. Webster, M.R.C.S.Eng., appointed Medical Officer of Health for the Vaynor and Penderyn Rural District. E. J. Weightman, M.B., C.M.Edin., appointed Medical Officer for the Workhouse and Children's Homes of the Middlesbrough Union. E. T. Whittaker, M.B., C.M.Edin., D.P.H.Camb., appointed Medical Officer of Health for the Wem Urban District. W. R. E. Williams, M.B., M.R.C.S., &c., appointed Assistant Resident Medical Officer to the Mill Road Infirmary, Liverpool.

Central London Throat and Ear Hospital.—The following appointments have been made: Assistant Surgeons—P. H. Abercrombie, M.D., C.M.; W. J. C. Nourse, F.R.C.S.E. Assistant Anæsthetist—A. Barford, M.D.

### BOOKS FOR NURSES.

**Domville's Manual for Hospital Nurses,** 8th edition ... 2/6
**Cullingworth's Monthly Nursing,** 4th edition... 1/6
**Hellier's Gynæcological Nursing...** ... 1/6
**Cuff's Lectures on Medicine to Nurses** ... 3/6
**Richmond's Antiseptic Principles for Nurses** 1/-
**Mayne's Short Dictionary of Medical Terms,** 2/6
**Chavasse's Advice to a Wife,** 14th edition, by Dr. FANCOURT BARNES ... 2/6
**Chavasse's Advice to a Mother,** 15th edition, by Dr. GEORGE CARPENTER .. ... 2/6

LONDON: J. & A. CHURCHILL, 7, Great Marlborough Street.

THE MOST NUTRITIOUS.
### EPPS'S
GRATEFUL—COMFORTING.
### COCOA
BREAKFAST AND SUPPER.

### THE NURSE'S REPORT BOOK.

Foolscap 4to., Black cover, **6d.** net. By Post 8d.

**Compiled by K. H. (Cert.)**

H. J. GLAISHER, 57, Wigmore Street, Cavendish Square, W.

28 & 29, SOUTHAMPTON STREET, STRAND, LONDON, W.C.

Accouchement Requisites.

Ambulance Cases.

### NOW READY,

A REPRINT OF

### A NURSING HANDBOOK

TOGETHER WITH S. MAW, SON, AND THOMPSON'S

### COMPLETE CATALOGUE OF NURSING REQUISITES.

The above work is now republished at a cost of 1s. per copy. Messrs. S. M., S., and T. have been compelled to make this charge on account of the extreme popularity of the work and the great expense incurred in its production.

***Post Free on receipt of 1s.***

### S. MAW, SON, and THOMPSON,

7 to 12, ALDERSGATE STREET, LONDON, E.C.

